# A Re-evaluation of Whether Non-monosynaptic Homonymous H Reflex Facilitation Tests Propriospinal Circuits

**DOI:** 10.3389/fnsys.2021.641816

**Published:** 2021-03-23

**Authors:** Karen M. Fisher, Stuart N. Baker

**Affiliations:** Henry Wellcome Building, Medical School, Newcastle University, Newcastle upon Tyne, United Kingdom

**Keywords:** propriospinal, H reflex, primate, median nerve, segmental interneurons

## Abstract

The C3–C4 propriospinal system is an important pathway mediating movement in cats; it contributes to movements in primates (including humans), and may have a role in recovery after lesion. Validated clinical tests of this system would find many applications, therefore we sought to test whether non-monosynaptic homonymous facilitation of the forearm flexor H reflex is mediated solely via a C3–C4 propriospinal pathway. In one anesthetized macaque monkey, median nerve stimulation elicited an H reflex in the flexor carpi radialis (FCR). Median nerve conditioning stimuli at sub-threshold intensities facilitated the H reflex, for inter-stimulus intervals up to 30 ms. Successive spinal surgical hemisections were then made. C2 lesion left the homonymous facilitation intact, suggesting mediation by spinal, not supraspinal pathways. Facilitation also remained after a second lesion at C5, indicating a major role for segmental (C7–C8) rather than propriospinal (C3–C4) interneurons. In separate experiments in five healthy human subjects, a threshold tracking approach assessed changes in peripheral axon excitability after conditioning stimulation. This was found to be enhanced up to 20 ms after the conditioning stimulus, and could partly, although not completely, underlie the H reflex facilitation seen. We conclude that homonymous facilitation of the H reflex in FCR can be produced by segmental spinal mechanisms, as well as by a supranormal period of nerve excitability. Unfortunately, this straightforward test cannot therefore be used for selective assessment of propriospinal circuits.

## Introduction

Motor commands can arrive at spinal motoneurones via a range of descending pathways. In humans, the corticospinal tract (CST) dominates, although other supraspinal and spinal structures can also contribute significantly to movement. Such multiplicity offers the potential to sub-serve some motor recovery following damage to the CST (Lawrence and Kuypers, 1968; Zaaimi et al., 2012).

One pathway whose functional importance was initially controversial in humans is the C3–C4 propriospinal system. This projection was first described comprehensively in cat. In this species, the C3–C4 propriospinal system provides a fast relay of motor commands from the motor cortex (M1) to forelimb motoneurons; it is especially important in visually guided reaching (Alstermark et al., 1981). Early lesion studies localized propriospinal interneurons to the C3–C4 segments in the spinal cord (Illert et al., 1977). These cells receive extensive convergent input from descending pathways (cortico-, rubro-, reticulo-, and tectospinal tracts) (Illert et al., 1977, 1981), as well as peripheral feedback during movement. Descending inputs provide monosynaptic excitation and disynaptic inhibition of C3–C4 propriospinal interneurons (Alstermark et al., 1984) which in turn directly excite or inhibit forelimb motoneurones. This profound convergence from multiple pathways at a pre-motoneuronal level allows optimization of motor commands.

The presence and role of propriospinal interneurones in primates has been widely debated over the past few decades. In contrast to the cat, primates have direct monosynaptic cortico-motoneuronal (CM) connections (Bortoff and Strick, 1993), which partially underlie the ability to perform dextrous hand movements (Porter and Lemon, 1993). Initial studies in anesthetized macaques reported very few disynaptic excitatory post-synaptic potentials (EPSPs) in motoneurons following corticospinal tract stimulation (Maier et al., 1998); this was supported by more indirect measures from single motor unit discharge in awake animals (Olivier et al., 2001). Subsequent work hypothesized that feedforward inhibition of propriospinal cells masks their actions following stimulation of the whole CST. Antagonizing this inhibition with strychnine injections in anesthetized animals revealed abundant disynaptic EPSPs which were abolished by spinal section at C5 but not at C2, suggesting a propriospinal substrate (Alstermark et al., 1999). More focal stimulation within the motor cortex induces later EPSPs which are consistent with disynaptic mediation without the use of strychnine (Witham et al., 2016). In awake monkey, lesions or genetically induced inactivation of C3–C4 propriospinal neurons suggest that they can partially mediate fine finger movements (Sasaki et al., 2004; Alstermark et al., 2011; Kinoshita et al., 2012; Sugiyama et al., 2013; Tohyama et al., 2017). Finally, injections of anatomical tracer substances in the hand representation of M1 reveal CST terminals in C3–C4 as well as in the cervical enlargement, consistent with a hypothesized role in hand function (Yoshino-Saito et al., 2010). This extensive work showed clearly that the propriospinal system makes an important contribution to primate motor control.

Non-invasive electrophysiological measures suggest that a similar C3–C4 propriospinal system exists in humans (Burke et al., 1994; Mazevet and Pierrot-Deseilligny, 1994; Pierrot-Deseilligny, 1996; Nicolas et al., 2001; Stinear and Byblow, 2004a,b; Pierrot-Deseilligny and Burke, 2012; Nakajima et al., 2017; Suzuki et al., 2017; Irie et al., 2020), and that it receives corticospinal (Gracies et al., 1994) and peripheral inputs (Malmgren and Pierrot-Deseilligny, 1988a,b). This work typically used the Hoffman reflex (H reflex) to measure motoneuron excitability. In the human upper limb, the H reflex is most easily elicited in the flexor carpi radialis (FCR) muscle following stimulation of the median nerve. One of the earliest studies investigated possible proprioceptive inputs to propriospinal interneurons by giving weak sub-threshold median nerve stimulation 2–20 ms before a median nerve shock which elicits the H reflex. This yielded an enhanced reflex response, suggesting convergence of inputs on the FCR motoneurons (Malmgren and Pierrot-Deseilligny, 1988a). A further facilitation could be generated if both ulnar and median nerves were activated as the conditioning stimulus. Based on the timing of these effects, it was argued that a propriospinal pathway is the most likely substrate.

The existence of non-invasive methods for assessment of the propriospinal system would assist greatly in determining the role these cells may play in neurological disease or recovery after damage (see, e.g., Mazevet et al., 2003 for an example of this approach). However, this is only possible if the proposed method can be shown to rely solely on propriospinal pathways. The papers which introduce such methods typically present only circumstantial evidence for a propriospinal contribution, and are admirably cautious in their conclusions. With non-invasive methods alone, it is impossible to exclude contributions from segmental or brainstem pathways, instead of or in addition to the hypothesized propriospinal circuit. The original papers in cat argued conclusively for propriospinal pathways using selective spinal lesions either above or below the C3–C4 segments (Illert et al., 1977), an approach which has more recently been replicated in monkey to argue for a propriospinal contribution to precise finger movements (Sasaki et al., 2004; Alstermark et al., 2011). Studies developing non-invasive methods have not hitherto validated them using a selective lesion approach.

In this paper, we investigate one phenomenon which has been claimed to result from propriospinal circuits in humans. We have chosen the homonymous facilitation of the FCR H reflex following prior conditioning with a weak median nerve shock, as this is a straightforward protocol using just a single stimulating electrode and which does not require the subject to produce voluntary activity. As such, it could find widespread application in studies of human disease. After replicating the findings from humans in anesthetized macaque monkey, we showed that similar results could be obtained after both C2 and C5 spinal hemisection. Our findings reveal that mechanisms other than C3–C4 propriospinal interneurons can generate homonymous H reflex facilitation, and that this unfortunately cannot be used as a definitive measure of propriospinal function.

## Materials and Methods

Results from a single anesthetized healthy adult female macaque monkey (weight 8.2 kg) are presented in this report. A further six animals were tested on similar protocols, but difficulties in eliciting a consistent and clearly measurable H reflex, anesthetic complications, or a failure to generate a sufficient lesion prevented useable findings in these monkeys. It should be noted that eliciting a clear H reflex in the FCR muscle can also be difficult in human subjects. All animals had previously participated in various unrelated studies, and were used in this experiment as a terminal procedure. All animal procedures were carried out in accordance with United Kingdom Home Office regulations (Animals in Scientific Procedures Act, 1986) and ethical approval was granted by the Animal Welfare and Ethical Review Board of Newcastle University.

### Surgical Preparation

Surgical and anesthetic procedures followed our previous work (Fisher et al., 2012; Witham et al., 2016; Aguiar and Baker, 2018). Initial surgery was performed under deep general anesthesia maintained with inhaled sevofluorane (1–2% in 100% O_2_) and supplemented with continuous intravenous infusion of alfentanil (5–8 μg/kg/h). Methylprednisolone was administered initially as a loading dose (30 mg/kg) and then infused continually throughout the experiment (5.4 mg/kg/h) to reduce oedema. Initial preparation included a tracheotomy, and insertion of a central arterial line via one carotid artery to measure blood pressure. A bladder catheter was inserted to drain urine. A tri-polar nerve cuff was implanted around the median nerve in the upper portion of the left arm. A laminectomy was performed to expose spinal segments C2–T1. The dura mater was removed to expose the spinal cord. The vertebral column was clamped at the high thoracic level and the head was fixed with the neck in a flexed position in a stereotaxic frame. Anesthesia was then switched to a combination of midazolam (0.5–0.8 mg/kg/h), ketamine (0.3–0.8 mg/kg/h) and alfentanil (13–23 μg/kg/h) during the electrophysiological recordings, as we have found that this combination yields more excitable spinal circuits. Physiological signs (arterial blood pressure, heart rate, pulse oximetry, capnography, peripheral and core temperature) were monitored throughout the procedures. Drug infusion rates were adjusted in response to both gradual and sudden changes in these parameters to ensure deep and stable anesthesia was maintained.

### Stimulation and Recording

Insulated stainless steel wires (Advent Research Materials, Oxford, United Kingdom, order number FE6320), bared for 1–2 mm at the tip, were inserted into the left FCR muscle using needles to record electromyogram (EMG; amplifier bandpass 30 Hz–2 kHz; gain 100; sampling rate 5 kSamples/s). Monopolar stimuli (0.5 ms width) were applied to the median nerve using a Digitimer DS4 stimulator (inter-stimulus interval 4 s), choosing one pair of the available three contacts in the nerve cuff to optimize the H reflex. Motor threshold was determined as the intensity at which an M wave appeared. The stimulus intensity was then adjusted to elicit a stable H reflex. This often proved difficult, as the short conduction distances in monkey mean that the H reflex overlaps the later part of the M wave; however, by adjusting the stimulus intensity, and repositioning the EMG recording wires, it was possible to obtain a measurable H reflex at onset latency around 7 ms, which is appropriate given estimates of conduction distances and known afferent and efferent conduction velocities (Cheney and Preston, 1976a,b).

A conditioning series was then recorded by preceding the median stimulus which elicited the H reflex with a conditioning stimulus with intensity set to 0.6× motor threshold; we verified that the conditioning stimulus given alone did not elicit an H reflex. The following 28 conditioning intervals were tested: 1–3 ms in steps of 0.5 ms, 4–20 ms in steps of 1 ms, 22–30 ms in steps of 2 ms. The inter-stimulus interval was 4 s; different intervals were randomly interleaved, together with the test stimulus alone. Ten responses to each condition-test interval, and 30 or 40 responses to the test stimulus alone were recorded. Data capture and stimulus delivery were controlled by a 1401 intelligent laboratory interface and Spike2 software (both Cambridge Electronic Design, Cambridge, United Kingdom).

### Spinal Cord Hemisection

Spinal cord hemisections were performed ipsilateral to the stimulation and recording sites, at the C2 or C5 segments, using a scalpel blade. Hemostasis was straightforwardly achieved by packing the cut region of the cord with Gelfoam. Following hemisection, further sets of condition-test intervals as described above were recorded.

### Histology

To determine the extent of the lesion, we carried out post-mortem analysis of the spinal cord. At the end of the experiment, the animal was perfused through the heart with phosphate buffered saline and formalin fixative. Blocks of spinal cord were cryoprotected in ascending concentrations of sucrose solution (final solution 30%), embedded in Tissue-Tek glue (Sakura Finetech, Thatcham, United Kingdom), frozen and sectioned (50 μm thickness) on a microtome. Sections were transferred onto slides and stained with cresyl violet. The sections were then digitized. Outlines of the section in which the lesion was maximal, together with a nearby complete section, were overlain in the figures to indicate the region destroyed.

### H Reflex Data Analysis

Analysis was carried out in the MATLAB programming environment using custom software. We measured the peak-to-peak amplitude of the H reflex in each single sweep. Results for a given inter-stimulus interval are presented as the mean amplitude of the conditioned responses as a percentage of the response to the test stimulus given alone. Error bars on plots are the standard error of the mean. The significance of any facilitation or suppression was determined from an unpaired *t*-test, comparing the single sweep values, with a threshold of *P* < 0.05.

### Nerve Threshold Changes in Healthy Human Subjects

Whilst examining results from all of the monkey experiments, we noticed that in some cases the M wave was changed by conditioning stimuli. To investigate how the threshold for nerve stimulation might modulate after a conditioning stimulus to the same nerve, we carried out a further set of experiments in five healthy human subjects. Studies were approved by the Ethics Committee of the Faculty of Medical Sciences, Newcastle University, and subjects provided informed written consent. Experiments conformed to the Declaration of Helsinki, except for pre-registration in a database.

Participants were seated and at rest throughout the testing period. EMG was recorded from the resting FCR muscle, using adhesive surface electrodes in a bipolar montage with the active contact positioned over the muscle belly (amplifier bandpass 30 Hz–2 kHz, gain 500, sampling rate 5 kSamples/s). Electrical stimulation of the median nerve in the arm was performed using a DS5 stimulator (Digitimer Ltd, Welwyn Garden City, United Kingdom) and a clinical-style stimulating electrode which was held firmly in place with a Velcro strap. The stimulus intensity was adjusted to produce a small but consistent M wave, just above motor threshold; denote this intensity T. The amplitude of this M wave was then measured.

We then used a stimulus protocol which interleaved trials with just the test stimulus (eliciting an M wave) with trials which preceded the test stimulus with a conditioning stimulus at a given interval. The intensity of the conditioning stimulus was fixed at 0.6xT, which was below motor threshold (i.e., did not produce an M wave). The intensity of the test stimulus was continually adjusted by a threshold tracking algorithm (Bostock et al., 1998) to maintain the M wave amplitude constant at the level measured initially. Trials on which the amplitude was smaller than the target led to an increase of the stimulus on the next trial; trials where a larger M wave than the target was elicited led to a reduced stimulus intensity on the next trial. Intensities were increased or decreased in 20 μA steps. Threshold tracking was performed separately for the trials with test stimulus alone, and those conditioned by a preceding sub-threshold stimulus. A total of 200 stimuli (100 with test stimulus alone, 100 with a conditioning stimulus, interleaved) were given at a particular inter-stimulus interval. This protocol was repeated for intervals between 1 and 32 ms. The average stimulus intensity in the last 50 stimuli, when the threshold tracking had converged, was used as an estimate of the nerve threshold. The ratio of the threshold following the conditioning stimulus to the threshold when only a test stimulus was given was calculated for each inter-stimulus interval.

## Results

### Spinal Hemisections in Monkey

**FIGURE 1:**
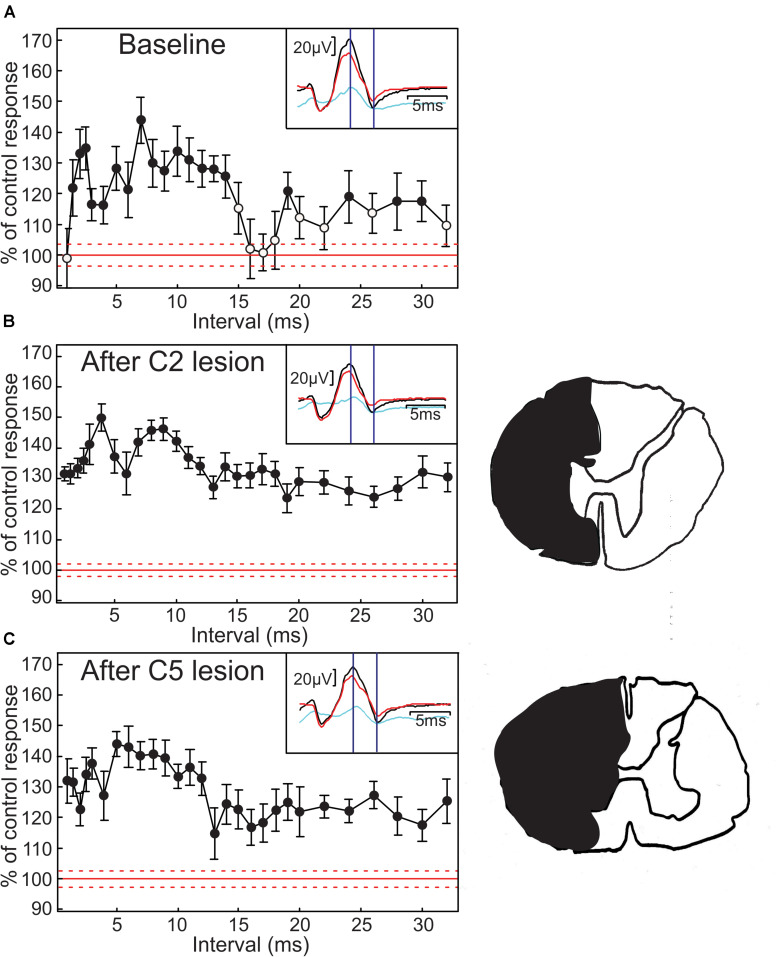
Modulation of H reflexes in anesthetized monkey. **(A)** modulation of H reflex as a function of condition-test interval. Reflex size is expressed as a percentage of the response following test stimulus alone. Error bars indicate SEM; filled symbols mark points significantly different from 100% (*P* < 0.05). Red line indicates the 100% level, with dotted lines marking the SEM of the test reflex size. Insets show responses to test only (red) and conditioned at an interval of 7 ms (black), with the difference between these responses plotted in light blue. Vertical lines indicate latencies used to measure H reflex amplitude. Test stimulus 1.87× motor threshold (MT), conditioning stimulus 0.6× MT. **(B)** H reflex facilitation profile, after ipsilateral hemisection at C2. **(C)** The same after ipsilateral C5 hemisection. Drawings on the right are traced from a photograph at the largest extent of the lesion in each case, indicating the region destroyed in black.

Figure 1A demonstrates the main effect which we have investigated in this report, measured in an anesthetized monkey. Each point plots the H reflex amplitude in the FCR muscle when conditioned by a preceding sub-threshold stimulus to the median nerve at different inter-stimulus intervals. Amplitudes are expressed as the percentage of the amplitude to the test stimulus alone. There was a long-lasting reflex facilitation. The first phase (2–15 ms intervals) was comparable to that reported by Malmgren and Pierrot-Deseilligny (1988a) in healthy human subjects. There was also a second, smaller phase of facilitation from 18 to 30 ms (Figure 1A). The insets to Figure 1A illustrates the recorded M wave and H reflex, and marks the points from which the peak-peak measurements of H reflex amplitude were made. As noted in section “Materials and Methods,” short conduction distances in monkey mean that these responses are not as clearly separated as in human, but two different components can be distinguished. Only the H reflex modulated with conditioning stimulation in this case, as is made clear by the difference plot (light blue).

**FIGURE 2:**
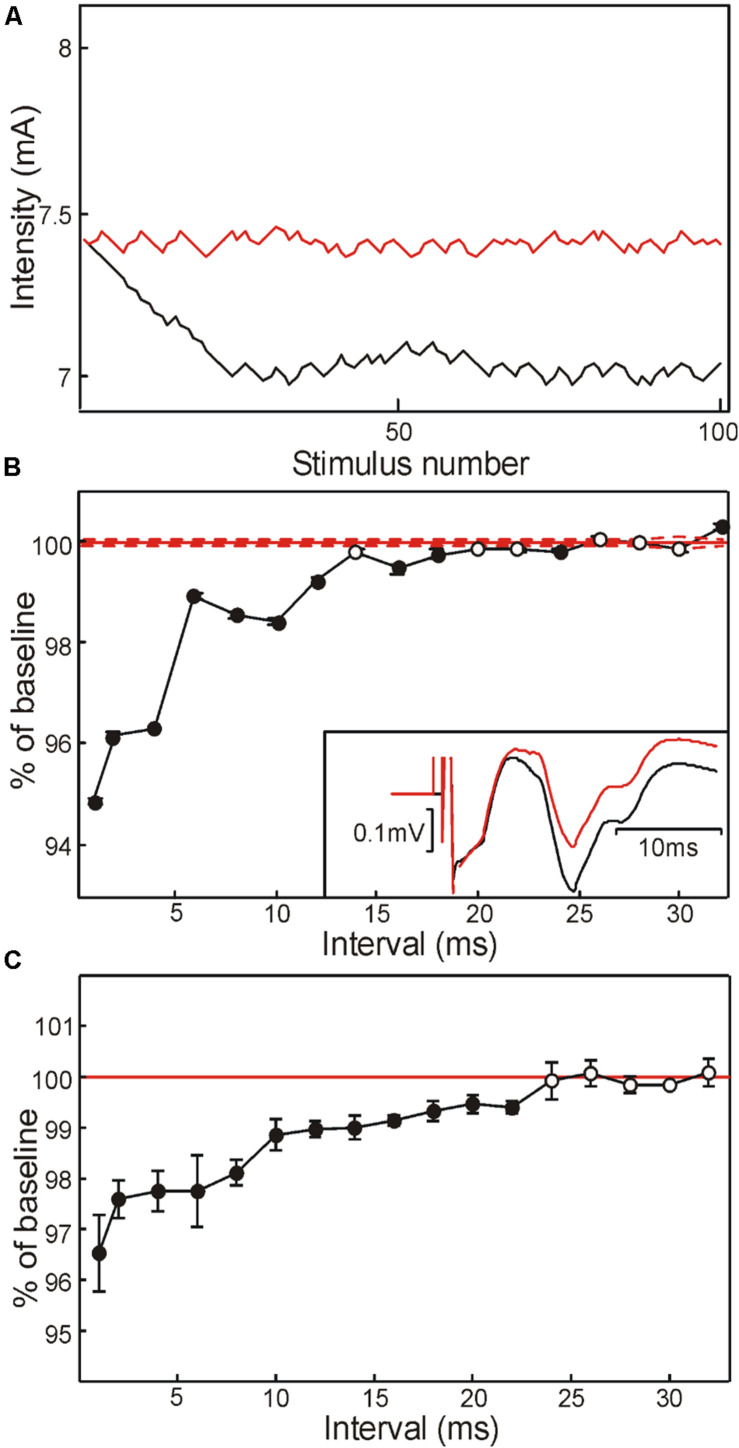
Modulation of axon threshold assessed using threshold tracking of M response in healthy human subjects. **(A)** Variation of stimulus intensity with successive stimuli, as the tracking algorithm attempted to match the target response size. Red trace, test stimulus alone; black trace, condition and test stimulus with interval of 1 ms. **(B)** Change in M wave threshold with different condition-test intervals, in a single subject. Threshold is expressed as a percentage of that measured following test stimulation alone (red). Points are the mean and SEM measured over the last 50 stimuli at a given interval, by which point the algorithm had converged [see panel **(A)**]. Inset illustrates averaged traces from this subject following test stimulus alone (black) and conditioned at a 1 ms interval (red). **(C)** Modulation in M wave threshold averaged across five healthy human subjects. In panels **(B,C)**, filled symbols indicate points significantly different from 100% (*P* < 0.05).

Next, we aimed to determine the neural circuits generating the H reflex facilitation. After gathering a control conditioning curve, we eliminated the effects of supraspinal inputs (e.g., from the reticulospinal and corticospinal tracts) by making a spinal lesion at the level of C2. The conditioning protocol was then repeated, and there was little change to the H reflex facilitation (Figure 2B). Reconstruction of the lesion (outline to right of Figure 2B) revealed that there was some spared intermediate gray matter together with a small portion of the white matter on the stimulated side. However, almost all descending inputs were abolished by this lesion; the continued facilitation in Figure 1B compared to Figure 1A strongly suggests that supraspinal pathways play little role in this effect.

Similar results were obtained following the second lesion, to the C5 segment of the spinal cord (Figure 2C). Again, the profile of facilitation remained very similar to that obtained before lesion. In this case, there was some sparing of the ventral white matter (outline to right of Figure 2C). However, as propriospinal interneurons project axons down the lateral funiculus, their influence should have been largely abolished. This result is consistent with a predominantly segmental origin of the H reflex facilitation.

### Nerve Threshold Changes

The M wave results from direct electrical stimulation of the motor axons; as such, it cannot be changed by fluctuations in neural circuitry, but reflects only the excitability of the axons. During experiments in other animals, it became apparent that the M waves sometimes also modulated their amplitude with conditioning stimulation. If the M wave modulates, this presumably indicates that the motor axon excitability was changed by the sub-threshold conditioning stimulus. Similar effects occurring in the group Ia afferents would lead to an enhanced sensory volley, which could partially explain the large H reflex produced at some intervals.

To investigate changes in axonal excitability in more detail, we used the threshold tracking paradigm as described in section “Materials and Methods.” Figure 2A shows how the stimulus intensity varied to produce an M wave of a fixed amplitude (0.32 mV), in one subject. The red trace shows the results when only the single stimulus whose intensity is plotted was given. The black trace shows the results when this stimulus was preceded by a 4.45 mA shock at an interval of 1 ms. The tracking algorithm progressively reduced the test stimulus intensity until the response amplitude reached the target. For the final 50 sweeps, the mean intensity required was 7.40 mA for the single shock, but 6.03 mA following the conditioning stimulus.

Figure 2B plots the threshold estimated in this way, as a function of inter-stimulus interval for a single subject. There was a significant reduction in threshold for almost all intervals below 20 ms (filled symbols, *P* < 0.05). Figure 2C presents an average across all five subjects tested on this paradigm. Tracked M wave amplitudes were between 0.32 and 0.61 mV (mean 0.43 mV). Similar to the results from Figure 2B, there was a significant reduction in threshold at short intervals, which returned to baseline by 24 ms.

## Discussion

We designed this study to probe whether homonymous facilitation of upper limb H reflexes in non-human primates and humans is mediated by the C3–4 propriospinal system. We were able to replicate in anesthetized monkey the homonymous facilitation previously reported by Malmgren and Pierrot-Deseilligny (1988a) in healthy humans.

Our findings following experimental spinal cord hemisection contradict previous suggestions that non-monosynaptic facilitation in humans is mediated by C3–C4 propriospinal fibers (Malmgren and Pierrot-Deseilligny, 1988a). Although we do not exclude the possibility that there is some contribution from this pathway, it is likely to be minimal, with the major role provided by other neural systems.

Lesions of the spinal cord at C2 will disrupt ascending and descending pathways. This would remove any reflex facilitation which was carried by a long-loop pathway. Examples might be corticospinal or reticulospinal responses to the sensory input, which could facilitate FCR motoneurons. In addition, there might be a more tonic effect of such a lesion: for example, the removal of descending input to spinal circuits might render them inexcitable and hence unable to generate reflex facilitation. The homonymous facilitation was clearly retained after the C2 lesion, suggesting that pathways above this level made little contribution.

The C5 lesions removed not only inputs from the major descending motor pathways, but also the influence of propriospinal interneurons which have their cell bodies in the C3–C4 spinal segments, and project axons down the lateral funiculus to the cervical enlargement. Once again, we were able to see preserved H reflex facilitation after C5 lesions. This clearly demonstrates that segmental circuits not involving systems rostral to the cervical enlargement are capable of mediating the effect.

Although we present data from only one animal, the existence of a single observation is sufficient to show that homonymous reflex facilitation can be generated by non-propriospinal pathways. We naturally cannot exclude some role for C3–C4 propriospinal cells, but this measure cannot form the basis of a test for the efficacy of propriospinal circuits.

It was of interest that at the shortest interval tested (1 ms), no facilitation was seen in the control condition (Figure 1A), but that facilitation appear at this earliest interval after both spinal lesions (Figures 1B,C). We can only speculate on the reasons for this change, but note that a complete spinal hemisection produces profound acute changes in segmental circuitry, including in reflexes (Sherrington, 1906). This could easily shift the balance between inhibitory and facilitatory interactions, leading to the appearance of facilitation as seen.

### Potential Confounding Factors

It is important to consider the role of drug interactions during studies in anesthetized animals. Reports in the literature demonstrate a mixed effect of anesthetic drugs on H reflexes. Sevofluorane administered in sub-anesthetic doses significantly reduces the amplitude of H reflexes in humans (Kammer et al., 2002); we, however, ensured that no inhalation anesthetic was used during our recordings. Although higher doses of sevoflurane were used for preparatory surgery, this is rapidly cleared and we would expect no residual effects. We did use ketamine (which antagonizes NMDA receptors) as part of the anesthetic mixture; this has been reported to enhance H reflexes (Bastron et al., 1972). Midazolam, which was also part of our mixture and which enhances GABA_*A*_ transmission, might be expected to depress H reflexes via activation of pre-synaptic inhibition. However, a previous report indicated that another benzodiazepine, diazepam, has no effect on the H reflex, although it does enhance paired-pulse depression (Crayton, 1980). Reassuringly it was possible to replicate the basic finding of homonymous facilitation under anesthesia. It is therefore unlikely that anesthesia materially affected our main conclusions.

### Proposed Pathways to Produce Non-monosynaptic H Reflex Facilitation

The finding that homonymous facilitation of the H reflex is retained even after C5 spinal hemisection indicates that systems below this level are capable of generating the effect. It is clear that part of the facilitation may arise from the properties of the nerve itself. After firing an action potential, there is a robust window of heightened excitability within peripheral axons which follows the relative refractory period. Studies in animals (Gasser and Erlanger, 1930; Gasser and Grundfest, 1936) and human subjects (Gilliatt and Willison, 1963; Ng et al., 1987; Kiernan et al., 1996; Kuwabara et al., 2000; Burke et al., 2001) have shown such supranormal excitability starts at approximately 3 ms and continues up to 15–20 ms after a spike. Our conditioning stimulus was sub-threshold for eliciting an overt H reflex, but will have stimulated many large diameter afferents, which will then have shown a post-spike supranormal period. However, if these axons were stimulated by the weak conditioning stimulus, they would also have been activated by the stronger test stimulus. A *post-spike* supranormal period cannot therefore generate an enhanced afferent volley (comprising more active fibers) to the test stimulus.

In addition to firing some axons, the conditioning stimulus will also depolarize some fibers below their spiking threshold; this could enhance their subsequent excitability. Prior studies by Shefner et al. (1996) and Chan et al. (2002) provided indirect evidence for supranormality after sub-threshold stimuli. This was investigated in more detail by Bostock et al. (2005), who showed that increased excitability lasted around 13 ms after a sub-threshold stimulus. In our data from Figure 2, the period of enhanced excitability following a sub-threshold stimulus lasted around 20 ms, broadly consistent with Bostock et al.’s study. A post-spike supranormal period occurs in sensory axons, although to a lesser extent than motor fibers (Kiernan et al., 1996), and it is hence likely that similar subthreshold effects also occur. The test stimulus would therefore recruit a greater number of fibers from the liminal fringe when it follows a weak conditioning stimulus than when it is given alone. The resulting enhanced afferent volley would produce a larger H reflex.

Although effects within sensory axons probably play a role, they are unlikely to be the sole cause of the homonymous H reflex facilitation. The changes in motor axon threshold seen following a sub-threshold stimulus were small, and decayed roughly exponentially (Figure 2). For intervals longer than 10 ms, there were threshold reductions of 1% or less. By contrast, the H reflex could show a long-lasting large (>20%) facilitation, with effects at 10–15 ms comparable to those at shorter intervals (Figure 1). It is likely therefore that there was also a central effect which enhanced motoneuron excitability and increased the H reflex response to the test stimulus.

Since the homonymous H reflex facilitation survives spinal hemisection at C5 level, spinal circuits close to the segmental location of the FCR motoneurons are likely to play a significant role. Primate spinal interneurons receive inputs from multiple descending motor pathways (Riddle and Baker, 2010) as well as from peripheral sources and can exert powerful effects on motoneurons (Perlmutter et al., 1998). Spinal circuits therefore contribute to long-latency stretch reflexes (Soteropoulos and Baker, 2020). The homonymous H reflex facilitation was previously suggested to arise from propriospinal systems because of a relatively long central delay, estimated at 3 ms (Malmgren and Pierrot-Deseilligny, 1988a). It was suggested that this could arise from the conduction time for impulses to travel to more rostral segments, and then back again.

The most accurate data on central conduction delays in human relate to fast corticospinal fibers. Similar measurements are available in monkey, and this can provide us with scale factors necessary to scale up other measures made only in monkey to humans, as follows. Central motor conduction time (CMCT) in the corticospinal tract from M1 to the cervical enlargement in human is around 7.5 ms (Jaiser et al., 2015). Standard CMCT measures include the delay for the cortico-motoneuronal synapse; assuming this to be 1 ms, this leaves 6.5 ms for axonal conduction time. In monkey, corticospinal conduction times from the medulla to the cervical enlargement are approximately half that from M1 (Edgley et al., 1990; Riddle et al., 2009; Fisher et al., 2015; Witham et al., 2016), and equal to ∼0.7 ms. The fastest conduction delay in the axons of propriospinal interneurons from C3 to C4 to the cervical enlargement is also 0.7 ms (Isa et al., 2006). A reasonable estimate of the conduction time in human propriospinal axons can therefore be made as half the CMCT, 6.5 ÷ 2 = 3.2 ms. Segments C3–C4 are approximately halfway between the medulla and the C7 segment where most FCR motoneurons are located (Jenny and Inukai, 1983). Assuming that ascending afferent axons conduct at similar velocities to the corticospinal tract, a reasonable estimate of the afferent delay from C7 to C3–C4 would be one quarter of the CMCT, 6.5 ÷ 4 = 1.6 ms. The total expected central delay for a propriospinal pathway can be estimated as the sum of delays in ascending afferents (1.6 ms), descending propriospinal axons (3.2 ms), and a synaptic delay (1 ms), which comes to 5.8 ms. Propriospinal delays should thus be larger than the 3 ms estimated by Malmgren and Pierrot-Deseilligny (1988a) for the central delay of the homonymous facilitation. Clearly such estimates have a degree of imprecision, but the available data are not unambiguously in support of a propriospinal pathway.

A long central delay could be generated by segmental circuitry in several ways. Firstly, it is possible that the pathway is oligo-, rather than di-synaptic, and hence that the extra central time simply relates to additional synaptic delays. Secondly, it may be that the interposed interneurons are shifted rostro-caudally relative to the FCR motoneurons, whilst remaining within the cervical enlargement. In cat lumbar cord, Bannatyne et al. (2009) showed that axonal projections of excitatory interneurons can be traced rostral and caudal to the cell body. Fibers traveling within the median nerve enter the dorsal roots of segments C5–T1 (Netter, 2014); there is thus the possibility of axonal conduction delays over two segments to reach the FCR motoneurons in C7. It is reasonable that spinal circuits within the cervical enlargement could generate the observed central delay.

## Conclusion

In this study, we have used spinal lesions to demonstrate that homonymous facilitation of the FCR H reflex can be generated by segmental, rather than propriospinal pathways. Selective lesions were used in the paper which first demonstrated the existence of a propriospinal system in cat (Illert et al., 1977); the method is attractive for the unambiguous information which it can provide concerning underlying pathways. Logically, even a single observation of surviving responses after a lesion indicates that the lesioned circuit is not required for the effect. Our results mean, unfortunately, that the straightforward measure of homonymous H reflex facilitation first introduced by Malmgren and Pierrot-Deseilligny (1988a) cannot be used to assess propriospinal circuits selectively. Several other non-invasive measures have been proposed to reflect propriospinal activity in humans (for comprehensive review, see Pierrot-Deseilligny and Burke, 2012). The present work in no way addresses to what extent those measures could be mediated by non-propriospinal pathways. Each specific method must be considered in isolation, and its selectivity for propriospinal pathways assessed independently, a task which remains for future studies.

## Data Availability Statement

The original contributions presented in the study are included in the article/supplementary material, further inquiries can be directed to the corresponding author.

## Ethics Statement

The studies involving human and animal participants were reviewed and approved by the Animal Welfare and Ethical Review Board of Newcastle University. The participants provided their written informed consent to participate in this study.

## Author Contributions

KF designed and performed all experiments, analyzed the data, wrote the first draft of the manuscript and contributed to revisions until the final version. SB designed and performed all experiments, analyzed the data, and contributed to revision of the manuscript until the final version. Both authors contributed to the article and approved the submitted version.

## Conflict of Interest

The authors declare that the research was conducted in the absence of any commercial or financial relationships that could be construed as a potential conflict of interest.
